# Fly immunity comes of age: The utility of *Drosophila* as a model for studying variation in immunosenescence

**DOI:** 10.3389/fragi.2022.1016962

**Published:** 2022-10-04

**Authors:** Mary-Kate Corbally, Jennifer C. Regan

**Affiliations:** Institute of Immunology and Infection Research, University of Edinburgh, Edinburgh, United Kingdom

**Keywords:** *Drosophila*, immunosenescence, immunity, ageing, natural variation

## 1 Introduction

### 1.1 The challenge of understanding individual variation in immunosenescence

The global demographic is shifting towards an aged population (United Nations, 2019). Yet, it is our oldest members of society that are the most vulnerable to infectious disease, reflecting immune system ageing, or “immunosenescence” (Glynn and Moss, 2020; Chen et al., 2021). Immunosenescence has two defining features: heightened susceptibility to pathogens, and increased systemic, basal inflammation (Franceschi, 2000; Zerofsky et al., 2005; Benayoun et al., 2019), which is strongly linked to the development of age-related diseases such as neurodegeneration and cancer (Franceschi et al., 2018; Rea, 2018). Understanding the mechanisms of immunosenescence could offer therapeutic potential in reducing age-associated morbidity (Neves and Sousa-Victor, 2020; Borgoni et al., 2021). However, individuals do not age at the same rate, with variation arising from genotype (Burger and Promislow, 2006; Belsky et al., 2015), including sex (Sampathkumar et al., 2020; Xirocostas et al., 2020; Bronikowski et al., 2022), and from the environment. While most mechanistic biology research ignores this diversity, largely due to the practicality of using single genotypes and sexes, individual variation will determine the response to therapeutics targeting ageing pathologies (Meyer et al., 2013; Fuselli, 2019). Here, we champion the use of *Drosophila melanogaster* as an ideal model for the early stages of an approach that aims to leverage population variation to understand immunosenescence and how to treat it. We briefly describe the rich history of this model system in the fields of the biology of ageing, biogerontology and immunology, and their conceptual and technical convergence on the fly model. We outline the unique potential of *Drosophila* in elucidating variation in immunosenescence, its underlying mechanisms and its treatments.

### 1.2 *Drosophila*’s central role in formulating evolutionary theories of ageing—why do organisms age?

*Drosophila* have proven pivotal in the synthesis and empirical testing of the evolutionary theories of ageing, which provide a conceptual framework within which to understand the biology of ageing. Experimental evolution experiments highlighted a trade-off between longevity and early-life fecundity (Rose and Charlesworth, 1981; Luckinbill and Clare, 1985; Rose, 1991). Such fitness trade-offs are commonly seen in insects (Flatt et al., 2013) and give credence to Williams’ theory of antagonistic pleiotropy (Williams, 1957). These trade-offs may rely on resource allocation between germline and “disposable” soma (Kirkwood, 1977), and are not a universal, but rather a contextual, *quid pro quo* that depends on environmental factors. The range of phenotypic expression of a genotype across varying environments is called phenotypic plasticity. It is an important source of natural variation for life history traits. This has been demonstrated in multiple settings, such as temperature and commensal status, using *Drosophila* (e.g., Lee et al., 2019; Huang et al., 2020). An influential evolutionary theory, the resource reallocation hypothesis, suggests that natural selection may favour phenotypically plastic responses in allocation, prioritising somatic maintenance during scarcity, and reproduction in replete settings (Shanley and Kirkwood, 2000; Regan, 2020). *Drosophila* has offered an easy-to-manipulate empirical platform to understand these phenotypically plastic responses to nutritional resources, and their associated trade-offs (Lee et al., 2008; Jensen et al., 2015; Lee, 2015; Zanco et al., 2021). These studies have served to shape our understanding of key evolutionary concepts on the consequence of reproductive investment, and phenotypically plastic responses to the environment, in determining rates of ageing.

### 1.3 *Drosophila*’s central role in biogerontology—how can we slow down ageing?

The goal of Biogerontology, born out of evolutionary biology, is to enhance healthy ageing by limiting age-associated multi-morbidity through therapeutic intervention. Key to this goal is a thorough understanding of the underlying molecular mechanisms that govern the ageing process. Ageing has been studied using flies for over a century (Hyde, 1913; Piper and Partridge, 2018). One of the observations fundamental to modern-day biogerontology was the remarkably beneficial effects of dietary restriction on ageing across taxa. The use of invertebrate model organisms, such as flies and worms, has given insight into the dietary macronutrients, genes and pathways underpinning this effect (Grandison et al., 2009; Piper and Partridge, 2018). These studies have not only implicated environment-sensing pathways like IIS (Clancy et al., 2001; Tatar et al., 2001), and TOR (Vellai et al., 2003; Kapahi, 2004), but also numerous longevity-associated pathways including Toll, Ras-ERK-ETS, AMPK, and Myc pathways (Piper and Partridge, 2018). The extension of lifespan through targeted manipulation of pathways such as TOR was initially demonstrated in invertebrates (Vellai et al., 2003), including *Drosophila* (Bjedov et al., 2010), before it was shown to be effective in mammals (Harrison et al., 2009).

### 1.4 *Drosophila*’s central role in immunology—the genetics of innate immunity

The famous intersection of genetic tractability with immunology following the identification of *Toll* as an immune signalling molecule in *Drosophila* (Lemaitre et al., 1996), and later characterisation of mammalian Toll-like pathogen-recognition receptors (Medzhitov et al., 1997), sealed *Drosophila*’s fate as an indispensable model system in innate immunity research. Indeed, there are multiple conserved immune components of the model. Immune signalling culminates in expression of antimicrobial peptides (AMPs) and immune effectors *via* conserved NF-kB-like transcription factors (Lemaitre and Hoffmann, 2007; Buchon et al., 2014). Haematopoiesis, controlled by conserved transcription factors (Evans et al., 2003), gives rise to macrophage-like hemocytes which share a common superfamily of phagocytic receptors with humans (Kurucz et al., 2007). Epithelial immunity, such as the gut, is governed by conserved immune signalling, regulates commensal interactions, and is structurally similar to that of humans (Liu et al., 2017).

### 1.5 Power to the fly: *Drosophila* as a nexus for immunity, evolution and biogerontology to understand variation in immunosenescence

**FIGURE 1:**
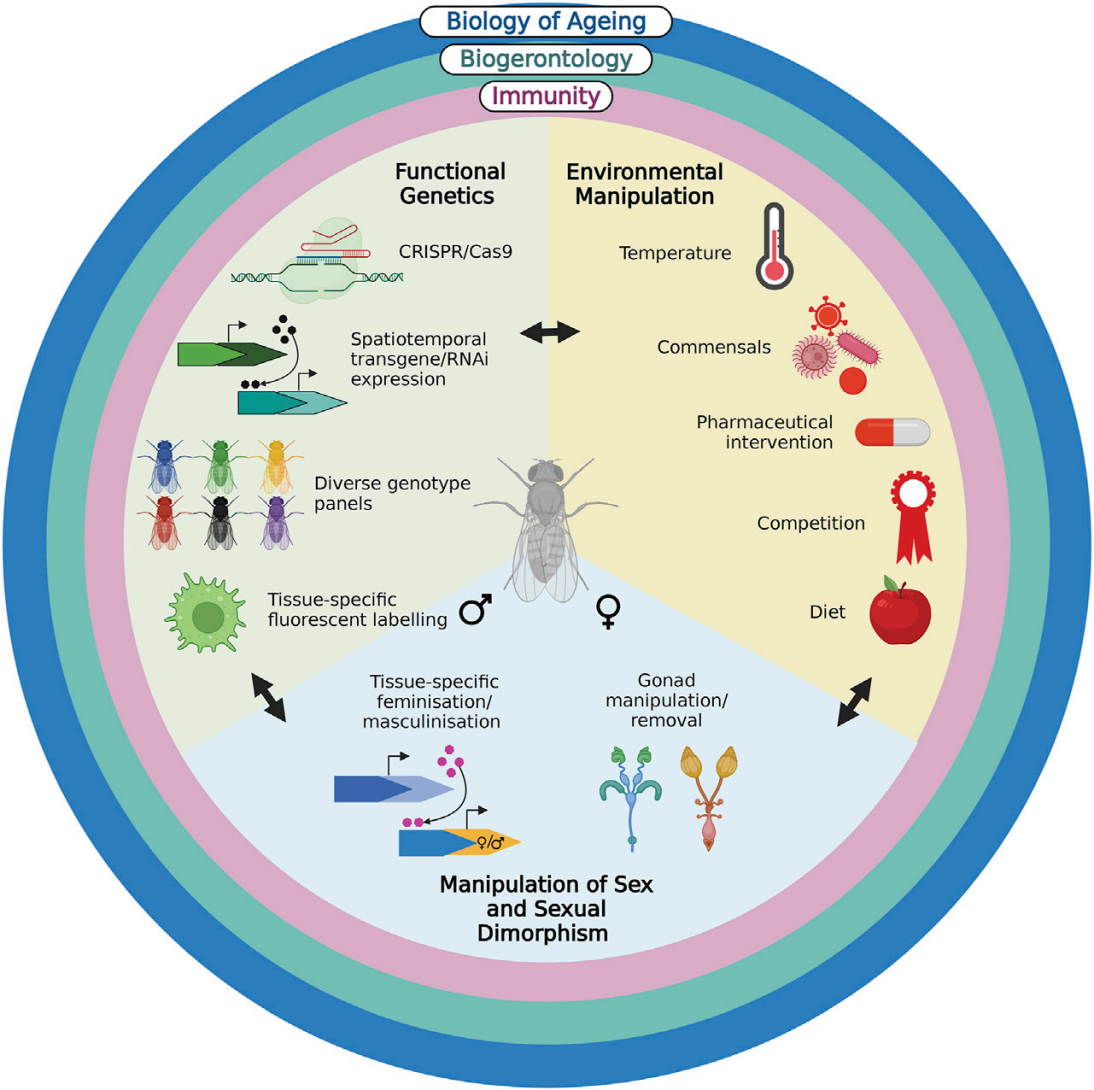
*Drosophila*’s rich history in the fields of evolutionary biology of ageing, biogerontology and immunity have endowed the model with multiple conceptual approaches and techniques. The functional genetic approaches, ease of environmental and sex manipulation render it invaluable in understanding natural variation in immunosenescence and its potential treatments. Created with BioRender.com.

The rich history of *Drosophila* as a model system, spanning more than a century, has produced biological insights too numerous to mention. Here, we have briefly detailed the contributions of the model to three, interconnected disciplines. The power, we argue, in using *Drosophila* as a model in immunosenescence, which shares many similarities with that of mammals and has been reviewed elsewhere (Flatt and Garschall, 2018; Min and Tatar, 2018; Sciambra and Chtarbanova, 2021), comes from the conceptual and technical convergence of these fields on the system (Figure 1). Coupling the genetic and technical approaches possible in *Drosophila* with an evolutionary biology approach to address different forms of variation in immune responses will not only provide mechanistic insights into immunosenescence, but will also guide our ultimate goal of treating it.

#### 1.5.1 Genetic variation

Genetic and phenotypic heterogeneity in immune responses are ubiquitous across species. Panels of fully-sequenced, isogenic lines derived from wild-caught *Drosophila* (Mackay, 2012; Grenier et al., 2015), or from extensive recombination (King, 2012), are an important community resource for understanding the genetic bases of complex phenotypic traits. These lines have been used to explore natural variation in, and genetics underpinning, susceptibility to pathogens. Susceptibility is determined by both resistance, which describes the ability to control pathogen burden (Wang et al., 2017; Palmer, 2018; Chapman et al., 2020) and disease tolerance (Howick and Lazzaro, 2017), which describes the ability to cope with infection and represents a less well-understood immune strategy (Soares et al., 2017; Schneider, 2021). *Drosophila* offers an ideal model to address the elusive processes determining disease tolerance, as they offer readily measurable fitness metrics and genetic tractability (e.g., Kutzer and Armitage, 2016; Duneau et al., 2017a; Gupta and Vale, 2017). Assessing variation in disease tolerance to *Providencia rettgeri* has implicated several biological processes, such as genes related to the endoplasmic reticulum (Howick and Lazzaro, 2017), which was further linked to tolerance in a transcriptomic analysis of infection response (Troha et al., 2018). These studies have granted some initial insight into the physiology underpinning this relatively elusive immune strategy, and genetic variation therein.

Use of isogenic panels could be extended to explore natural variation and plasticity in immunosenescence and the immune strategies affected by age. The role for declines in disease tolerance, and its variation, in driving immunosenescence is currently a black box, largely due to our lack of understanding of the mechanisms underpinning tolerance. There is a dearth of genotypically high-powered experiments assessing genetic variation in age-related immune dysregulation, despite prevalent genotype-by-age interactions (Lesser et al., 2006; Felix et al., 2012).

By performing genome wide association analysis (GWAS; e.g., King, 2012; Mackay, 2012) when assessing such variation in immunosenescence among the isogenic panels, candidate genes and pathways could potentially be identified, and ultimately validated. The ability to conditionally manipulate virtually any gene in the fly genome, through the spatiotemporal control of transgene expression *via* binary genetic systems (Brand and Perrimon, 1993; Osterwalder et al., 2001), makes validation of candidate genes possible in high throughput. These tools grant the model relative ease in assessing canonical and non-canonical components of immune defence and their impact on immunosenescence over ageing. Additionally, *Drosophila* are tractable to reverse genetic knock-out approaches using CRISPR/Cas9 technology, as has been demonstrated in the functional assessment of individual AMPs (Hanson et al., 2019). Recently, the potential of such CRISPR lines in the functional assessment of AMPs over ageing has been demonstrated. While ageing is associated with expansion of the AMP repertoire and loss of specificity, possibly indicative of immune dysregulation (Shit et al., 2022), their collective role in regulating microbiome dysbiosis was shown to be a critical determinant of lifespan (Hanson and Lemaitre, 2022), the control of which has previously been linked to lifespan and ageing pathologies (Guo et al., 2014; Clark et al., 2015; Li et al., 2016) Finally, fluorescent labelling of immune tissues, such as hemocytes (e.g., Sanchez Bosch et al., 2019; Krejčová, 2019; Chakrabarti and Visweswariah, 2020; Kierdorf, 2020; Coates, 2021), opens the possibility to assess their contribution to immune, and systemic, ageing.

Flies are highly amenable to experimental evolution, which when coupled with genomic sequencing, allows inferences to be made about the genetic response to a dominant selection pressure, such as delayed reproduction or longevity (McHugh and Burke, 2022). This technique can be used to explore trade-offs between immunity and life-history traits (McKean et al., 2008; Shahrestani et al., 2021), or lack thereof (Faria, 2015; Gupta, 2016). In lines selected for longevity, an improvement of measurable immune responses was observed (Fabian, 2018), whereas selection for resistance against a specific fungal pathogen was costly to lifespan (Shahrestani et al., 2021). It is possible, and remains to be tested, that improved early immunity has detrimental effects on the rate of immunosenescence, ultimately affecting ageing.

#### 1.5.2 Plasticity in immunity

Immunology research has historically focused on the genetics underpinning immune responses within a controlled environment, but rarely assesses reaction norms under varying conditions (Martin, 2021). *Drosophila* has offered insight into such immune plasticity, including genotype-by-temperature (Lazzaro et al., 2008), and genotype-by-diet interactions using isogenic lines. The observed effects of diet on resistance (Unckless et al., 2015) underscore the importance of nutrition in determining immune responses (for e.g., Galenza, 2016; Ponton et al., 2020). Similarly, disease tolerance plasticity was observed in a diet- and pathogen-specific manner (Howick and Lazzaro, 2014; Kutzer and Armitage, 2016). Through the assessment of genotype-by-environment interactions, these investigations have highlighted an intrinsic link between immunity and metabolism, as has been confirmed elsewhere, for example, the induction of immune-response genes downstream of IIS and TOR nutrient-sensing pathways independently of Toll or Imd signalling (Becker et al., 2010; Varma et al., 2014). Thus, environmental factors are an important source of variation in immunity, as predicted by evolutionary theory.

Little is known about phenotypic plasticity in the manifestation of immunosenescence. Yet, the question “*what kind of environments accelerate or slow down immunosenescence?*” is crucial to an evolutionary biology approach in treating immune decline. Plasticity in immune responses in the face of varying environments early in life (Lazzaro and Little, 2009; Leech, 2019) is likely to have consequences on immunosenescence. While such plasticity is often ignored, or difficult to address, as in mouse studies (Martin, 2021), assessing the expression of immune responses across differing environments in flies is relatively tenable (McKean and Nunney, 2005; Lazzaro et al., 2008; Unckless et al., 2015).

Pharmacological intervention represents a highly relevant example of environmental manipulation, and we have barely scratched the surface in understanding genetic variation underpinning responses to geroprotective treatments (but see Lind, 2017; Rohde et al., 2021). Through robust genetic validation, flies have provided mechanistic insight into the action of multiple potential geroprotective drugs (for e.g., Bjedov et al., 2010; Slack et al., 2015; Castillo-Quan et al., 2019). *Drosophila* is the ideal candidate model to move towards high-powered studies addressing variation in response to either environmental (e.g., dietary restriction) or pharmacological (e.g., therapeutic mTOR attenuation) interventions targeting ageing, including immunosenescence. The crucial next step in biogerontology is to capture individual variation in responses to therapeutics, which, in contrast to mammalian systems, can be tested with relative ease in *Drosophila*.

#### 1.5.3 Sex bias in immunity

Sex differences in immune responses, which have been observed across taxa (Kelly et al., 2018; Metcalf et al., 2020), are prevalent in *Drosophila* infectious disease models (Belmonte et al., 2020). Sex differences in the immune response appear contextual in *Drosophila* (Belmonte et al., 2020), mirroring the lack of ubiquitous sex differences across taxa (Kelly et al., 2018). Sex differences in infection outcome are likely to be dictated by a complex interplay of environmental factors, pathogen-specific consequences of infection, and the different life-histories of the sexes. Notwithstanding this complexity, fruit flies have offered mechanistic insight into pathogen-specific, dimorphic responses to infection, such as sex differences in the Toll pathway (D. Duneau et al., 2017a; Shahrestani et al., 2018), and sex-by-genotype interactions determining viral load and transmission potential of *Drosophila* C virus (Siva-Jothy and Vale, 2021).

The inclusion of both sexes when using isogenic, transgenic or outbred *Drosophila* lines will invariably provide insight into sex differences in immunosenescence. In particular, examining dimorphism across genetic panels could address the interaction of sex and ageing on immunosenescence. Furthermore, the ability to remove the gonads prior to next generation sequencing facilitates the examination of somatic, sex differential gene expression, as was done to compare the immune response to *P. rettgeri* (Duneau et al., 2017b). Coupling the cell autonomous sex determination system of *Drosophila* (Salz and Erickson, 2010) with their robust genetic toolkit allows the manipulation of sex in a tissue-specific manner, as has been demonstrated in exploration of sexual dimorphisms in physiology (Hudry et al., 2016; Millington et al., 2021) and in the infection response and pathology of the ageing gut (Regan et al., 2016). This offers an unparalleled system to tease apart how tissue-specific and systemic sex differences contribute to individual variation in immunosenescence. Considering the conserved dimorphism in both immune function and responses to drugs that delay ageing (Bjedov et al., 2010; Arriola Apelo and Lamming, 2016; Bitto et al., 2016; Regan et al., 2016; Regan et al., 2021; Strong et al., 2020); *Drosophila* genetic techniques could be leveraged for the early stages of developing sex-optimised drug treatments for immunosenescence.

## 2 Conclusion

Immune decline over ageing is linked to age-associated morbidity through increased pathogen susceptibility and dysregulated inflammation. Yet, immunosenescence remains relatively elusive where individual-to-individual variation in the onset and extent is virtually unknown, potentially determining responses to treatments targeting the decline. Steps to capturing population diversity in immune responses, arising from genotype, phenotypic plasticity and sex, discussed here, would provide mechanistic insight if applied to immunosenescence. We argue that *Drosophila* is uniquely situated to address the challenge of variation in ageing. Its vast history as a model in the fields of evolutionary biology, biogerontology, and immunity offers a foundation of knowledge about the species and a matchless system of tractable genetics, statistical power, and environmental manipulation. Using *Drosophila* to initiate an evolutionary biology approach to immunosenescence would address population diversity and, ultimately, aid the development of personalised therapeutics.
